# 860 The Use of Human Amniotic Membrane Allograft in the Treatment of Indeterminate Depth Thermal Injury

**DOI:** 10.1093/jbcr/iraf019.391

**Published:** 2025-04-01

**Authors:** Louis Ferrari, Hussein ElGhandour, Suzanne Osborn, Karen Richey, Kevin Foster

**Affiliations:** Diane & Bruce Halle Arizona Burn Center; Diane & Bruce Halle Arizona Burn Center; Diane & Bruce Halle Arizona Burn Center; Diane & Bruce Halle Arizona Burn Center; Diane & Bruce Halle Arizona Burn Center

## Abstract

**Introduction:**

Technological advances in the field of wound care have been exponential over the past decade. According to the National Institute of Health, there are 76 dermal substitutes for treatment of wounds. One such advance is the emergence of human amniotic membrane allograft. The application of this technology in the care of burn patients is of great interest and there is a paucity of independent research examining its efficacy and efficiency. The purpose of this study was to evaluate human amniotic membrane allograft (HAMA) as treatment of partial thickness burns.

**Methods:**

This is a retrospective case series of patients of all ages, with partial thickness thermal injury, treated over a two-year period. All were treated with tangential excisional debridement and application of HAMA. Patients with mixed depth or full thickness injury were excluded. Basic demographic, injury, surgical and hospital data were collected. Descriptive statistics were calculated.

**Results:**

A total of 26 patients were treated with HAMA, the mean age was 36.9 years (range 1-80) with the majority being male (n= 17, 65%). Non-Hispanic Caucasians constituted the majority (n= 13, 50%) followed by Hispanic Caucasians (n=6, 23%). The most common cause of injury was flame (n= 9, 35%), the average total body surface area (TBSA) burn was 12.5% (range 2-72%). The mean total square cm treated per patient was 478 cm2 (range 30-1810 cm2) representing an average of 24% (range 22%-31%) of the total burn area per patient. The average charge per patient was $14,684.42 or $37.62 per cm2 treated. Autografting was not required for the majority of patients (n=22, 85%). Only one patient required a second treatment with HAMA. The mean length of stay was 19.8 days (range 2-79 days) or 1.87 days per % TBSA (range 0.4-5.8 days per % TBSA).

**Conclusions:**

Human amniotic membrane allograft is likely a viable treatment paradigm for medium to deep- partial thickness thermal injury. This independent review has revealed HAMA’s potential to avoid split thickness skin graft closure in this population. A prospective, comparative study is needed to examine possible reductions in morbidity and improved functional outcomes.

**Applicability of Research to Practice:**

The use of Human Amniotic Membrane Allograft should be considered a viable treatment of appropriate depth partial thickness thermal injury, where need for split thickness skin graft closure remains unclear.

**Funding for the Study:**

N/A

